# The Impacts of Domestication and Breeding on Nitrogen Fixation Symbiosis in Legumes

**DOI:** 10.3389/fgene.2020.00973

**Published:** 2020-08-18

**Authors:** Jinge Liu, Xiaocheng Yu, Qiulin Qin, Randy D. Dinkins, Hongyan Zhu

**Affiliations:** ^1^Department of Plant and Soil Sciences, University of Kentucky, Lexington, KY, United States; ^2^Forage-Animal Production Research Unit, United States Department of Agriculture-Agricultural Research Service, Lexington, KY, United States

**Keywords:** legume, nodulation, nitrogen fixation, symbiosis, domestication, rhizobia

## Abstract

Legumes are the second most important family of crop plants. One defining feature of legumes is their unique ability to establish a nitrogen-fixing root nodule symbiosis with soil bacteria known as rhizobia. Since domestication from their wild relatives, crop legumes have been under intensive breeding to improve yield and other agronomic traits but with little attention paid to the belowground symbiosis traits. Theoretical models predict that domestication and breeding processes, coupled with high−input agricultural practices, might have reduced the capacity of crop legumes to achieve their full potential of nitrogen fixation symbiosis. Testing this prediction requires characterizing symbiosis traits in wild and breeding populations under both natural and cultivated environments using genetic, genomic, and ecological approaches. However, very few experimental studies have been dedicated to this area of research. Here, we review how legumes regulate their interactions with soil rhizobia and how domestication, breeding and agricultural practices might have affected nodulation capacity, nitrogen fixation efficiency, and the composition and function of rhizobial community. We also provide a perspective on how to improve legume-rhizobial symbiosis in sustainable agricultural systems.

## Introduction

Nitrogen availability is a limiting factor for plant growth. Modern agriculture is heavily dependent on utilization of nitrogen fertilizers to sustain crop production, which is not only expensive but also contributes to severe environmental pollution and global warming. Legumes, however, are able to make their own nitrogen fertilizer through forming a root-nodule symbiosis with nitrogen-fixing soil bacteria collectively known as rhizobia. Inside nodule cells, the rhizobial symbionts find an environmental niche that enables them to convert atmospheric nitrogen to ammonia. The bacteria supply fixed nitrogen to the plant and the plant provides bacteria with carbohydrates derived from photosynthesis. This mutualistic relationship is overridingly important in biosphere nitrogen cycle and plays a critical role in maintaining agricultural sustainability.

Establishment of the legume-rhizobia mutualism is a complex process (Oldroyd et al., 2011). It begins with a cross-kingdom molecular dialogue, leading to altered development of both symbiotic partners. Starting in the rhizosphere, perception of legume root exudates (flavonoids) by the bacterium activates a specific set of bacterial nodulation (Nod) genes, leading to synthesis and secretion of a highly specific signal known as Nod factors or lipochitooligosaccharides. Perception of Nod factors by the plant LysM receptors induces a variety of host responses, including root hair curling, entry of the bacteria into the root hairs, induced expression of the host symbiotic genes, and cortical cell divisions that collectively culminate in the formation of rhizobia-infected root nodules. Different legumes vary in bacterial infection mode (root hair vs. crack/intercellular infection), nodule development (determinate vs. indeterminate), and bacterial differentiation inside the nodule cells (terminal vs. non-terminal). However, recent phylogenomic studies suggested a single evolutionary innovation of nodulation originated in a common ancestor of the so called “nitrogen-fixing clade” around 110 million years ago (Werner et al., 2014; Griesmann et al., 2018; van Velzen et al., 2018, 2019).

Conversion of nitrogen gas to ammonia is catalyzed by the rhizobial enzyme complex known as nitrogenase. This reaction is an energy-demanding process, requiring a large amount of adenosine triphosphates (ATPs) generated from aerobic metabolism of plant photosynthates. Paradoxically, however, the nitrogenase enzyme is exquisitely sensitive to, and irreversibly inactivated by, free oxygen. This conflict is perfectly resolved in the root nodule where oxygen concentration is buffered by the binding of oxygen with leghemoglobin (Terpolilli et al., 2012; Downie, 2014). To balance the trade-off and maximize the benefits of nitrogen fixation, legume hosts have evolved complex genetic mechanisms to tightly control nodule numbers or to block nodulation when sufficient nitrogen is available in the soil (Krusell et al., 2002; Searle et al., 2003; Nishida and Suzaki, 2018; Nishida et al., 2018; Ferguson et al., 2019). In particular, in autoregulation of nodulation, rhizobial infection activates the transcriptional regulator NIN that in turn triggers a systemic long-range signaling cascade between roots and shoots, resulting in inhibition of further nodule formation; under high soil nitrate conditions, a homologous NIN-Like Protein (NLP) is activated, which integrates into the autoregulation pathway to suppress nodulation and nitrogen fixation.

In contrast to wild species widely distributed in natural environments, modern varieties are developed for high input farming systems, thus deceasing their dependence on biological nitrogen fixation. It has been predicted that this dramatic transition from the wild to cultivation might have resulted in selection of genotypes that are more reliant on soil nitrogen and thus less able to take advantage of symbiotic nitrogen fixation, even though the symbiosis property is universally retained (Werner et al., 2015; Turcotte et al., 2017; Porter and Sachs, 2020). Such genetic shifts can be the consequence of relaxed selection on symbiosis traits, leading to altered host-bacteria compatibility, genotype-dependent root and soil microbial community, and the host’s ability to discriminate between high-efficient and low-efficient bacterial partners (Kiers et al., 2003, 2007; Westhoek et al., 2017; Porter and Sachs, 2020). Here, we review how domestication, breeding and agricultural practices might have affected the symbiosis traits in legumes and also provide a perspective on how such knowledge can be used for development of strategies to improve symbiotic nitrogen fixation in sustainable agriculture. It is noteworthy that the potential impacts of domestication on symbiosis discussed in this review are not independent but interrelated.

## Effect of Domestication and Breeding on Host-Rhizobial Compatibility

The legume-rhizobial symbiosis is a highly specific interaction, such that particular legume species/genotypes form an efficient symbiosis with only a specific set of rhizobial species/strains (Wang et al., 2012, 2018). Discordance can occur at various phases of symbiosis development. Incompatibility occurring at the beginning stages of the interaction can block bacterial infection and nodule organogenesis, while incompatibility taking place at later stages of the nodule development can result in development of infected nodules incapable of fixing nitrogen (Yang et al., 2010, 2017; Liu et al., 2014; Tang et al., 2016; Wang et al., 2017). Genetic control of symbiotic specificity is complex, involving a wide range of host and bacterial genes with various modes of action. In most legumes, rhizobial infection and nodule organogenesis requires host-specific recognition of bacterially derived Nod factors, and this recognition defines the basis of host-symbiont specificity (Geurts and Bisseling, 2002). While Nod factor perception is sufficient to cause nodule primordium formation, bacterial infection is also modulated by rhizobial effectors and microbe-associated molecular patterns (Fraysse et al., 2003; Deakin and Broughton, 2009; Kawaharada et al., 2015). Therefore, symbiosis development requires the bacteria to evade or overcome effector- and pattern-triggered immunity (Benezech et al., 2020).

The effect of domestication and breeding on host-rhizobial compatibility has been evaluated only in a handful of experiments (Table 1). Mutch and Young (2004) investigated host-range evolution for a population of *Rhizobium leguminosarum* strains sampled from nodules of pea (*Pisum sativum*), broad bean (*Vicia faba)* and several closely related wild species grown in a natural soil. Nodulation assays using a diverse panel of 80 isolates demonstrated that only 34% of the isolates were able to nodulate broad bean, while 89% were able to nodulate the wild species, suggesting that the domesticated legumes are less promiscuous in their interaction with the indigenous soil strains as compared to the wild legumes. Similarly, a genomic survey of rhizobia sampled from nodules of cultivated chickpea (*Cicer arietinum*) and its wild ancestor (*Cicer reticulatum*) showed that the wild species were associated with a more diverse *Mesorhizobium* population than cultivated chickpea (Kim et al., 2014). It was also reported that a higher abundance of *Bradyrhizobium* strains was present in the rhizosphere of wild species of soybeans (*Glycine soja*) than that of cultivated soybeans (*Glycine max*) (Chang et al., 2019). However, the conclusions drawn from these small-scale studies need to be further evaluated, because significant variation could also exist between different genotypes within the same wild or cultivated species. It is noteworthy that host range alone cannot dictate a cooperative advantage. Although reduced host range might limit nitrogen fixation if compatible strains are unavailable in a given soil, a more frequent scenario is that endemic strains are able to competitively nodulate the host legumes but with low nitrogen fixation efficiency. In this latter situation, excluding nodulation with the less efficient indigenous strains will increase the chances of the host to nodulate with more mutualistic symbiotic partners or added rhizobial inoculants.

**TABLE 1:** Significant examples of experimental studies on the effects of domestication and breeding on root nodule symbiosis in legumes.

**Potential impact**	**Legume host**	**References**	**Conclusion**
Symbiosis compatibility	Broad bean (*V. faba)* Chickpea (*C. arietinum*) Soybeans (*G. max*)	Mutch and Young (2004) Kim et al. (2014) Chang et al. (2019)	The cultivated species are less promiscuous in their interaction with indigenous symbionts than the wild legumes.
	Soybeans (*G. max*)	Devine (1985) Devine and Kuykendall (1996) Yang et al. (2010) Tang et al. (2016) Sugawara et al. (2019)	Analysis of frequencies of *Rj2*, *Rfg1*, and *Rj4* alleles in wild and cultivated soybeans did not reveal strong selection associated with domestication.
Coevolution of rhizobia with domestication	Common bean (*P. vulgaris)* Chickpea (*C. arietinum*)	Aguilar et al. (2004) Greenlon et al. (2019)	Both dispersal and novel adapted strains could have evolved with the spread of the domesticated legumes. Dispersed symbionts can transfer symbiosis genes into local strains that quickly became adapted.
Root-associated microbiome	Common bean (*P. vulgaris)* Soybeans (*G. max*)	Pérez-Jaramillo et al. (2016, 2018, 2019) Han et al. (2020)	There were significant differences in rhizosphere microbiome composition between domesticated plants and their wild relatives. These differences might have affected legume-rhizobial symbiosis in an indirect manner.
Partner choice and symbiosis capacity	Soybean (*G. max*) Pea (*P. sativum*) alfalfa (*Medicago sativa*)	Kiers et al. (2007) Provorov and Tikhonovich (2003)	The wild legumes and old varieties performed better than the advanced cultivars in terms of symbiotic nitrogen fixation, suggesting that the legume’s natural capability to distinguish between less- and high-efficient strains might be disrupted during domestication bottlenecks or breeding process.
	Soybeans (*G. max*)	Muñoz et al. (2016)	Analysis of 31 cultivated and 17 wild soybeans suggested that nitrogen fixation capacity was enhanced by soybean domestication.

A number of strain-specific nodulation-restrictive genes have been cloned in soybeans (e.g., *Rj2*, *Rfg1*, and *Rj4*) (Yang et al., 2010; Tang et al., 2016). These genes control rhizobial infection in the same manner as “gene-for-gene” resistance against pathogens. In particular, *Rj2* and *Rfg1* are allelic genes encoding nucleotide-binding/leucine-rich repeats (NLR) proteins that confer resistance to different groups of *Bradyrhizobium japonicum* and *Sinorhizobium fredii* strains (Yang et al., 2010). *Rj4* encodes a thaumatin-like defense-response protein that restricts infection by specific strains of *Bradyrhizobium elkanii* (Tang et al., 2016). The function of these genes is reliant on the rhizobial type III secretion system and its secreted effectors (Faruque et al., 2015; Sugawara et al., 2018). These studies revealed that effector-triggered immunity play a central role in the regulation of nodulation specificity.

The nodulation-restrictive alleles occur in both wild and cultivated populations, suggesting that these seemingly unfavorable alleles are retained during domestication and breeding processes. This could happen due to a lack of selection, or more likely because of balancing selection under the scenario that the same alleles could also confer resistance against the pathogens that secrete effectors homologous to those secreted by rhizobia (Deakin and Broughton, 2009). Alternatively, these alleles might have been maintained during domestication and breeding because they may confer an advantage for the host to interact with high-efficient strains and to exclude nodulation with less efficient ones, which is the case of the soybean *Rj4* allele that prevents nodulation by many strains of *B. japonicum* and *B. elkanii* (Devine and Kuykendall, 1996). *B. elkanii* is a poor symbiotic partner of soybeans because of its low nitrogen-fixation efficiency and its production of rhizobitoxine, a compound that induces chlorosis in the host plant. Thus, cultivars with the *Rj4* genotype are favorable in soils where the *B. elkanii* population is dominant. A survey of over 500 accessions of *G. soja* established an *Rj4* allele frequency of 63%, which is similar to that estimated from a cultivated soybean population sampled from Southeast Asia (66%) (Devine and Kuykendall, 1996). The *Rj4* allele is less frequent for cultivars sampled from East Asia (27%) and South Asia (16%). The geographic patterns suggested coevolution of host and bacteria in determining symbiotic compatibilities, because in Southeast Asia, warm climate provides an environment conducive to strains of *B. elkanii*. The *Rj2* allele is also present in both *G. soja* and *G*. *max*, with an allele frequency of 7.7% and 5.4%, respectively, based on genotyping of 1,324 cultivated and 259 wild soybean accessions, showing that the *Rj2* allele has been maintained during the domestication and breeding processes (Sugawara et al., 2019). In contrast, the *Rfg1* allele occurred with a much higher frequency (0.44) in a *G. max* population consisting of 285 genotypes from Asian countries (Devine, 1985). Even though the *Rfg1* allele frequency has not been broadly surveyed in *G. soja*, the distribution of the *Rfg1* (0.44) and *rfg1* (0.56) allele frequencies in cultivated soybeans suggests that these alleles has not been subjected to strong selection.

## Coevolution of Rhizobia With Legume Domestication

Legume hosts play a dominant role in interaction with rhizobia, and the soil rhizobial community has a strong influence on the symbiosis capacity of the plant hosts. The mutualism is established as a result of a coadaptation process between the symbiotic partners, which is also affected by environmental and climate variables and agricultural practices (Fox et al., 2007; Weese et al., 2015; Regus et al., 2017). A survey of *Rhizobium etli* strains that nodulate common beans (*Phaseolus vulgaris*) from the three putative bean domestication centers identified polymorphisms in the common nodulation gene *nod*C that are correlated with their geographical origins (Aguilar et al., 2004). Cross-inoculating wild beans from the three centers with these strains revealed preferential nodulation of beans by geographically related *R. etli* lineages. This observation was further supported by co-inoculation assays, which demonstrated that plants were almost exclusively nodulated by strains isolated from their host region, suggesting coevolution of the symbiotic partners in the host domestication centers.

**FIGURE 1:**
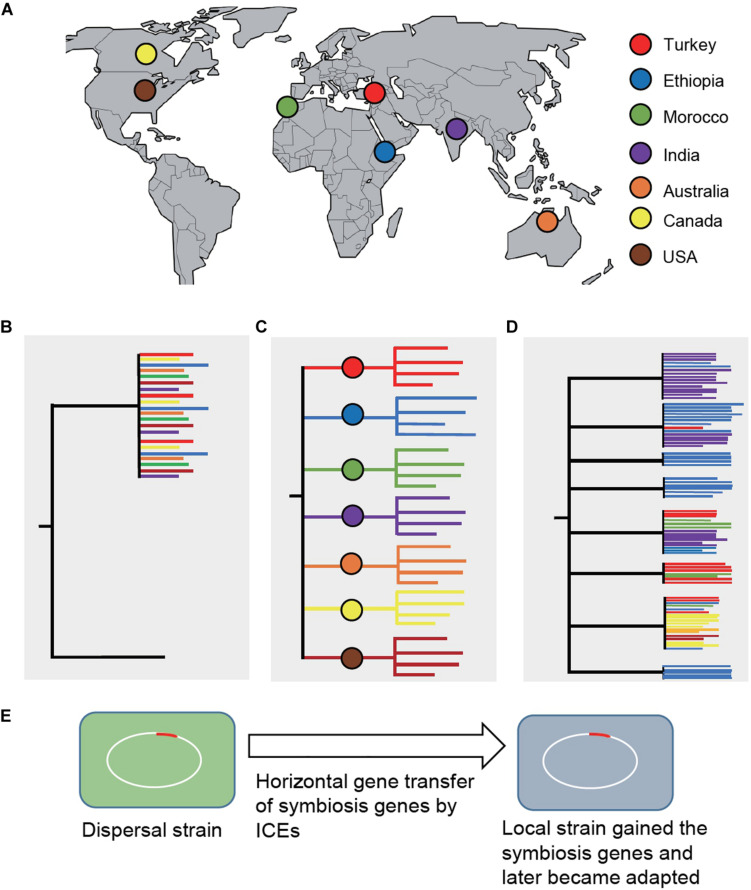
Phylogenetic analysis of *Mesorhizobium* strains sampled across the chickpea’s native and introduced ranges (Greenlon et al., 2019). **(A)** Geographic locations (countries) where the strains were sampled, including the chickpea’s domestication center, Turkey. **(B)** A phylogenetic diagram showing clustering of bacterial strains from the native and introduced ranges under the dispersal (co-introduction) model. **(C)** A phylogenetic diagram showing that bacterial strains would cluster based on their geographic locations and show a biogeographical pattern if the domesticated plants acquired novel symbionts in their introduced locations. **(D)** If both co-introduction and co-adaptation happened, the tree would show mixed patterns of **(A)** and **(B)**, which is the case revealed by the study of chickpea’s symbionts (Greenlon et al., 2019). Trees are not drawn in scale. **(E)** Strains in the introduced ranges might have acquired symbiosis genes from dispersed strains and subsequently became adapted and outcompeted the dispersal strains. Chromosomal symbiosis genes in *Mesorhizobium* are encoded on mobile integrative and conjugative elements (ICEs). ICEs can excise from the chromosome that can transfer and integrate into the chromosome of a recipient strain.

An intriguing question is how legume domestication might have driven the parallel evolution of rhizobial symbionts. Assuming that the wild relatives evolved a symbiosis with distinct bacteria, then after domestication and subsequent distribution to new environments, the domesticated crops either brought their natural symbionts with them (a process called dispersal), began to adapt to novel symbionts encountered at their new locations, or both (Bouznif et al., 2019). This question can be addressed by phylogenetic analysis of nodulating rhizobia across the crop’s native and introduced ranges. Dispersal would entail clustering of bacterial strains from the native and introduced ranges, otherwise bacterial strains would cluster based on their geographic locations and show a biogeographical pattern. A recent comprehensive study in chickpea has provided significant insights into this outstanding question (Greenlon et al., 2019; Figure 1), which is discussed below.

Chickpea (*C. arietinum*) was domesticated from the wild species *C. reticulatum* about 11,000 years ago in modern-day southeastern Turkey (von Wettberg et al., 2018). Since then, chickpea was distributed throughout the world, first in Middle East and Mediterranean basin, later reaching the Indian subcontinent and Ethiopia, and presently widely grown in countries where high-input agricultural practices are adopted, including Canada, United States, and Australia. This well-known domestication history of chickpea along with its global distribution provided an ideal system to evaluate the effect of plant domestication on the evolution of its symbiotic partners. For this purpose, rhizobial symbionts were sampled from nodules collected from cultivated chickpea and its wild relatives grown in multiple geographic locations representative of the majority of chickpea’s agricultural and natural ranges, including the plant’s center of origin in the southeastern Turkey. Phylogenomic analysis of over 1,000 draft whole-genome sequences revealed *Mesorhizobium* as the single bacterial genus consisting of a diverse panel of taxa that form symbiosis with chickpea, supporting that the wild relatives did evolve for symbiosis with specific bacterial partners.

Beyond chickpea’s native range, across regions where chickpea has been cultivated without applying specific rhizobial inoculants, the majority of root nodule bacteria are phylogenetically distinct from those derived from nodules of the wild relatives. Furthermore, phylogenetic diversity of the bacterial strains correlated with their geographic origins, mirroring edaphic and environmental factors (primarily the soil type and latitude), suggesting that the bacteria that dominate each location are likely adapted to the environmental conditions in those locations. Despite being diverse at the nucleotide level and in terms of genome structure, the genes associated with symbiosis share a high-level of gene synteny and sequence similarity to those found in chickpea’s natural symbionts, implying that chickpea’s coevolved symbionts dispersed along with the crop. In particular, the clade 5A consists of broad geographic distribution of strains that are closely related to those natural symbionts at wild chickpea’s center of origin. Taken together, both dispersal and novel adapted strains could have evolved with the spread of the domesticated chickpea. In this scenario, dispersed symbionts can transfer symbiosis genes into local strains that quickly became adapted and outcompeted the dispersal strains.

It was determined that evolutionarily divergent and geographically isolated bacterial taxa share a common set of integrative conjugative elements (ICEs) that encode the major functions of the symbiosis. The spread of the symbiosis ICEs is fixed in the microbe that originated at the crop’s center of origin. The relatedness of these ICEs is associated with both geographic distance and the evolutionary history of the background genome, parallel with the chickpea crop’s domestication and distribution. These ICEs could be a driving force to achieve increased diversity of compatible bacterial species by facilitating horizontal transfer of symbiosis genes across diverse *Mesorhizobium* taxa.

## Effect of Domestication on Root-Associated Microbiome

Plant microbiomes, particularly the rhizosphere microbiota, have been extensively studied in recent years using community profiling and omics analyses, but fewer studies have been carried out on legume microbiomes, especially during symbiosis development (Guttman et al., 2014; Hacquard et al., 2015; Müller et al., 2016). The root nodule symbiosis was long thought to be a highly specific binary plant-microbe interaction where the compatible nitrogen-fixing soil bacterium is recruited by the host. It has now become increasingly evident that root nodules contain not only nitrogen-fixing bacteria but many other bacterial species as well, some of which possess growth-promoting activities (Zgadzaj et al., 2015; Martínez-Hidalgo and Hirsch, 2017; Poole et al., 2018). Zgadzaj et al. (2016) compared root-associated microbiomes, including those from the root and rhizosphere, between *Lotus japonicus* wild type and nodulation-impaired mutant plants. This work revealed that the root-associated microbiomes are influenced by the host’s ability to accommodate symbiotic bacteria. For example, when symbiosis is lost, at least six different abundant bacterial orders become almost undetectable in the root; instead, some of these bacterial orders accumulate in the rhizosphere, suggesting that these bacteria are no longer able to enter the root. It is conceivable that these bacteria are involved in nodulation processes and symbiosis as helper bacteria, although it cannot be excluded that some of these bacteria are opportunists that have nothing to do with the actual symbiosis but hijack the symbiosis pathways to gain entry to the root. Nonetheless, these findings strongly suggest that the legume-rhizobia symbiosis is not just mediated by the rhizobia inside legume nodules, but also engages symbiosis-linked microbial communities that together may influence plant health, productivity, and adaption to biotic and abiotic stresses. Supporting this notion, Han et al. (2020) analyzed the composition and relationship of soybean rhizosphere microbiota in three types of soils and showed that the rhizosphere community differed significantly across different soils. Interestingly, the presence of some rhizosphere microbes was associated with the composition of *Bradyrhizobium* and *Sinorhizobium* bacteria in nodules; in particular, the *Bacillus cereus* group specifically promoted or suppressed the growth of *Sinorhizobium* and *Bradyrhizobium* bacteria, respectively, and alleviated the effects of saline–alkali conditions on the nodulation by *Sinorhizobium* strains and their colonization in nodules. This study demonstrated a crucial role of the bacterial microbiota in shaping the root nodule symbiosis in soybeans, suggesting that symbiotic capacity can be improved through the use of synthetic bacterial communities.

The impact of plant domestication and habitat expansion on root-associated microbiomes is not well understood. Several studies reported differences in rhizosphere microbiome composition between domesticated plants and their wild relatives (Zachow et al., 2014; Bulgarelli et al., 2015; Pérez-Jaramillo et al., 2016, 2018; Leff et al., 2017). In the legume common bean (*P. vulgaris*), Pérez-Jaramillo et al. (2017) reported that the rhizosphere of wild relatives was enriched in bacterial taxa from the phylum Bacteroidetes, while the rhizosphere of modern bean varieties was dominated by bacterial families belonging to the Actinobacteria and Proteobacteria, and that this compositional shift was associated with plant genotypes as well as root structure. Subsequently, the same set of common bean accessions representing a trajectory from wild to modern were grown both in a native soil and in an agricultural soil and their rhizosphere bacterial communities were profiled and compared (Pérez-Jaramillo et al., 2019). The study showed that the transition of common bean from a native soil to an agricultural soil led to a gain, rather a depletion, of rhizobacterial diversity. In particular, a small number of low-abundant bacterial genera were found only in the native soil and associated with only the rhizosphere of wild bean accessions, suggesting that these bacteria were depleted as a consequence of domestication and habitat expansion. In contrast, a higher number of bacterial taxa were found in the agricultural soil but not in the native soil, suggesting that the depleted bacterial genera in the agricultural soil were replenished with new bacterial taxa, many of which were highly abundant in the rhizosphere of all the common bean accessions. However, it is unknown whether such compositional changes in rhizosphere microbiomes have an impact on plant performance and whether such an observation is universal if more plant genotypes and soil types are tested. Unfortunately, all the above-mentioned experiments did not make a connection with symbiosis traits.

## Effect of Domestication and Breeding on Partner Choice and Symbiosis Capacity

Studies of binary legume-rhizobial interactions revealed tremendous variation in nitrogen fixation efficiency between different plant-rhizobial combinations. In particular, the same bacterial strain can form a highly efficient symbiosis with a given host but a low-efficient interaction with another, and no single host genotypes or rhizobial strains are consistently associated with the best nitrogen fixation performance. The failed or low-efficient partnership is often due to genetic incompatibility due to a lack of coadaptation between the interacting partners that negatively affects bacterial differentiation and survival inside the nodule cells. Thus, the host’s ability to choose best-matching partners from a pool of compatible rhizobia is key to improve the gain of nitrogen fixation.

It was hypothesized that natural hosts (wild species) might have evolved signaling mechanisms to preferentially partner with more mutualistic symbionts and to disadvantage less-cooperative ones (Younginger and Friesen, 2019). Evolutionary models predict that such natural surveillance capability can be disrupted during domestication bottlenecks or breeding process because of: (i) trading-offs between partner quality and agricultural traits as a result of negative trait association or antagonistic gene pleiotropy, such as defense versus symbiosis (Chen et al., 2017; Wood et al., 2018) or (ii) relaxed selection against symbiosis disruption in nitrogen-rich soils owing to a lack of observable fitness costs over disrupted symbiosis (Porter and Sachs, 2020). For example, when plants are grown in high-input agricultural soils, genotypes with reduced symbiosis capability are likely to have higher yields because they can freely get sufficient nitrogen from soil without need to allocate photosynthates to rhizobia for symbiosis development. However, very few experimental studies were carried out to test the hypothesis. Kiers et al. (2007) evaluated six soybean cultivars, representing 60 years of breeding, by infection with a mixture of effective and ineffective rhizobial strains. The experiments showed that older cultivars performed better than newer cultivars in terms of seed production, and this difference mirrored the relative fitness of effective and ineffective strains in their nodules, suggesting that the newer cultivars have reduced their capability to defend against less-efficient symbiotic partners. Provorov and Tikhonovich (2003) also observed that the wild-growing populations and local varieties of pea and alfalfa performed better than the advanced cultivars in terms of symbiotic nitrogen fixation.

However, Muñoz et al. (2016) reported that nitrogen fixation capacity was enhanced by soybean domestication. For their experiments, 31 cultivated and 17 wild soybeans were assayed for their nitrogen fixation efficiency by inoculation with the slow-growing *B. japonicum* strain USDA110 or the fast-growing *S. fredii* strain CCBAU45436. The results showed that cultivated soybeans overall out-performed wild soybeans in nitrogen fixation, as indicated by total nodule fresh weight, nodule number, total nitrogen, and total ureides accumulation (in soybeans, fixed nitrogen is first converted into ureides, which are exported out of nodules and transported to shoots, where they are catabolized). The lower nitrogen fixation efficiency of wild species appeared to be associated with higher abundance of polyhydroxybutyrate (PHB) bodies in bacteroids. PHB is an energy-rich compound that increases fitness of rhizobia but represents a net cost to the host, implying a trade-off between PHB accumulation and nitrogen fixation. Furthermore, genetic mapping in a recombinant inbred population derived from a cross between a cultivated and a wild parent identified several quantitative trait loci (QTLs) for total ureides and total nodule fresh weight. Association analysis revealed single nucleotide polymorphisms (SNPs) that are associated with these QTLs and these SNPs exhibited a low level of diversity in cultivated soybean, typical for domestication-related traits.

Despite inconsistent observations from different experiments, it is clear that there exists significant variation in symbiosis capacity between different plant genotypes in both wild and cultivated legume populations. This provides an opportunity for researchers to identify genetic loci/alleles that control the variation and integrate the genetic information into plant improvement through marker-assisted selection or gene editing.

## Conclusion and Perspective

Nitrogen fixation is dependent on the availability of the legume host’s bacterial partners in a given soil and the host’s ability to selectively interact with the most mutualistic partners from a group of compatible indigenous strains. In intensively managed agriculture systems, legume crops are often provided specific inoculants, but inoculation can fail if the added strains are not compatible with a given variety or easily displaced by endemic strains that are highly competitive for nodulation but less efficient for nitrogen fixation. Furthermore, the inoculant can become unstable over time because of gene loss and frequent horizontal gene transfer between soil bacteria (Brambilla et al., 2018). Thus, it is important for researchers to engineer or screen for elite rhizobia that are both competitive for nodulation and capable of high rates of nitrogen fixation, while also maintaining long-term stability in the intended soil environments (Checcucci et al., 2017; Westhoek et al., 2017). Most recently, a high-throughput screening method has been developed that allows for simultaneous measurement of both competitiveness and nitrogen fixation effectiveness of rhizobia at the single nodule level (Mendoza-Suárez et al., 2020); this technology provides the potential to revolutionize the search for super competitive and highly effective rhizobial symbionts.

It is equally important to improve host’s ability to choose and cooperate with the best mutualistic symbionts. For this purpose, genetic and genomic approaches should be employed to identify genes and alleles through harnessing abundant genetic and phenotypic variation present in the wild species and cultivated varieties. Lastly, understanding the effects of rhizosphere bacterial community on symbiotic capacity will provide an additional tool to manipulate the legume-rhizobial symbiosis. These coordinated strategies will yield the optimal combinations of partners’ genotypes and associated microbial helpers, ultimately leading to improvement of biological nitrogen fixation in sustainable agriculture.

## Author Contributions

All authors listed have made a substantial, direct and intellectual contribution to the work, and approved it for publication.

## Conflict of Interest

The authors declare that the research was conducted in the absence of any commercial or financial relationships that could be construed as a potential conflict of interest.
